# Dental prosthesis use and subsequent functional capacity decline among older adults: a three-year cohort study based on self-reported measures from the JAGES

**DOI:** 10.3389/froh.2026.1784871

**Published:** 2026-05-07

**Authors:** Mari Nakagawa, Kenji Takeuchi, Taro Kusama, Hazem Abbas, Katsunori Kondo, Hiroshi Egusa, Ken Osaka

**Affiliations:** 1 Division of Molecular and Regenerative Prosthodontics, Tohoku University Graduate School of Dentistry, Sendai, Japan; 2 Department of International and Community Oral Health, Tohoku University Graduate School of Dentistry, Sendai, Japan; 3 Division of Statistics and Data Science, Liaison Center for Innovative Dentistry, Tohoku University Graduate School of Dentistry, Sendai, Japan; 4 Department of Community Building for Well-being, Center for Preventive Medical Sciences, Chiba University, Chiba, Japan; 5 Institute for Health Economics and Policy, Research Department, Association for Health Economics Research and Social Insurance and Welfare, Tokyo, Japan

**Keywords:** aging, cognitive function, cohort study, functional capacity, prosthesis

## Abstract

**Background:**

Functional capacity (FC) is a key determinant of healthy aging. Oral health deterioration, particularly tooth loss, has been associated with FC decline among older adults. Although dental prostheses may mitigate functional deterioration by restoring oral function, longitudinal evidence on their protective effects on FC, especially across specific domains, remains limited. This study investigated the association between dental prosthesis use and the decline in overall and domain-specific FC among older Japanese adults experiencing tooth loss.

**Methods:**

We conducted a 3-year prospective cohort study using data from the Japan Gerontological Evaluation Study (JAGES). Participants were living independently older adults aged ≥65 years with <20 teeth at baseline (2016), followed up in 2019. Dental prosthesis use (removable dentures, fixed prostheses, and/or implants) was assessed by self-report at baseline and dichotomized as use vs. non-use. FC was measured using the Tokyo Metropolitan Institute of Gerontology Index of Competence (TMIG-IC), including total score and subdomains: instrumental activities of daily living (IADL), intellectual activity, and social role. FC decline was defined as a decrease of ≥1 point in the TMIG-IC score during follow-up. Modified Poisson regression with robust variance was used to estimate the relative risks (RRs) and 95% confidence intervals (CIs), adjusting for sociodemographic factors, health behaviors, comorbidities, and number of remaining teeth.

**Results:**

Among 22,632 eligible participants (mean age at baseline: 74.0 years, SD = 5.7; 51.4% male), 7,573 participants (33.5%) experienced a decline in total FC. Dental prosthesis use was significantly associated with a lower risk of decline in the total TMIG-IC score [RR = 0.91 (95% CI: 0.86–0.97)] compared with no use. For the TMIG-IC subdomain, we observed a significant association between dental prosthesis use and a lower risk of decline in intellectual activity [RR = 0.87 (95% CI: 0.80–0.96)].

**Conclusions:**

This large-scale cohort study demonstrated that the use of dental prostheses was associated with a reduced risk of FC decline in older adults, particularly in the intellectual activity domain. Because prosthesis use and FC were assessed by self-reported and residual confounding may remain, further studies are warranted to clarify the potential role of prosthodontic rehabilitation in maintaining FC in later life.

## 1 Introduction

In aging societies such as Japan, not only physical and cognitive functions but also social functions, such as interaction with others, are important factors to extend healthy life expectancy (1, 2). The concept of functional capacity (FC), which consists of the three domains of instrumental activities of daily living (IADL), intellectual activities, and social roles, has garnered attention as an assessment item to evaluate these functions (3). Tokyo Metropolitan Institute of Gerontology Index of Competence (TMIG-IC), one of the tools to assess FC, comprises 13 items: 5 items for instrumental independence, 4 items for intellectual activities, and 4 items for social roles, and is used as a scale to evaluate the abilities necessary for independent daily living at home (3). In a study using TMIG-IC, it was reported that the category with the lowest scores in the domains of instrumental independence, intellectual activity, and social role was significantly associated with all-cause mortality and long-term care requirement (4).

Oral health deterioration has been associated with a decline in FC. A previous study involving Japanese older adults reported that individuals with 0–19 teeth had lower TMIG-IC scores than did those with 20 or more teeth (5). Particularly, the level of disability in the edentulous group was reported to be comparable to that of those with a history of stroke (6). Furthermore, another study with a small sample size showed that those with fewer teeth and no history of denture use had particularly low TMIG-IC scores and often followed a trajectory of further functional decline by age 80 compared with the group that had higher TMIG-IC scores (7). These findings suggested that denture use may contribute to maintaining a higher level of FC (7). However, previous observational studies may be subject to confounding and selection bias, as socially active individuals often have better overall health and greater access to dental care. Reverse causality may also be present, because individuals with better functional status may be more likely to obtain or use dental prostheses.

Dental prostheses are intended to restore oral function after tooth loss. However, their effectiveness in improving masticatory performance and oral function may vary depending on the type of prosthesis, the degree of tooth loss, and individual adaptation to the prosthesis. Previous studies have also suggested that different types of prosthetic rehabilitation may have varying associations with health-related quality of life and depressive symptoms among older adults with tooth loss (8, 9). Nevertheless, the presence of a prosthesis does not necessarily indicate adequate prosthetic function or regular use in daily life. Consequently, the potential protective role of dental prosthesis use on the maintenance of FC remains unclear, and longitudinal evidence examining this association is limited.

Therefore, this study aimed to examine the association between dental prosthesis use and subsequent decline in overall and domain-specific FC as assessed by the TMIG-IC among Japanese older adults. We hypothesized that dental prosthesis use would be associated with a lower risk of FC decline among older adults. To address potential biases observed in previous studies, we employed a longitudinal cohort design and adjusted for a range of socioeconomic and health-related confounders.

## 2 Materials and methods

### 2.1 Study design and population

**Figure 1:**
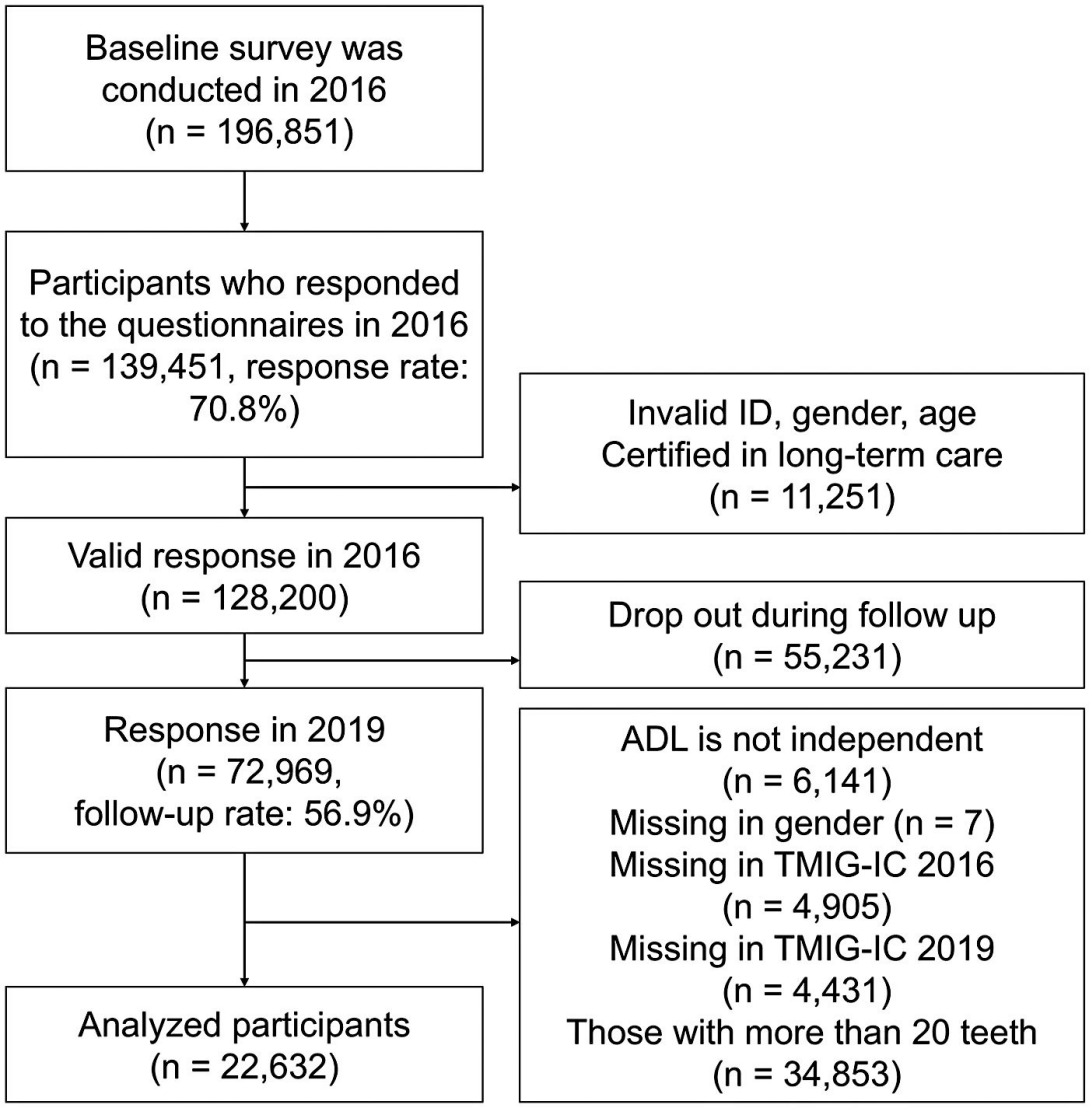
Flowchart of participant inclusion (*n* = 22,632).

The present study was a three-year follow-up cohort study based on a self-reported questionnaire. We used data from the Japan Gerontological Evaluation Study (JAGES), which is an ongoing cohort study targeting functionally independent older adults aged ≥65 years in Japan (10). The baseline survey was conducted in 28 municipalities from October to December 2016, and a follow-up survey was conducted from November 2019 to January 2020. The questionnaires were sent by mail and retrieved if the participants consented. Among the 196,851 targeted participants, 139,451 responded to the questionnaires in the baseline survey (response rate: 70.8%) (Figure 1). We were able to follow 72,969 participants in the follow-up survey (follow-up rate: 56.9%). We targeted patients with <20 teeth to examine the impact of dental prosthesis use on FC decline (11, 12).

### 2.2 Measurement

#### 2.2.1 Exposure variable

The exposure variable was the dental prosthesis use at baseline. We asked the following question: “Do you wear removable dentures or fixed prostheses or have dental implants?” The answers were “No,” “removable dentures,” “fixed prostheses,” and “dental implants.” We dichotomized this variable into “prosthesis use” or “non-use.” For the sensitivity analysis, we conducted detailed analyses using each type of dental prosthesis as an exposure variable (no use, removable dentures, fixed prostheses, implants, and use of more than two types of dental prostheses).

#### 2.2.2 Outcome variable

The outcome variable was the decline in total FC and the subdomains of FC (such as IADL, intellectual activity, and social role) from 2016 to 2019. FC was assessed using the TMIG-IC score (3). The changes in TMIG-IC scores have been evaluated over time to assess functional decline. The TMIG-IC questionnaire is presented in the Supplementary Text. Questions 1–5 were related to IADL, 6–9 to intellectual activity, and 10–13 to social role. The answer to each question was either “Yes (able to do)” or “No (unable)” and were scored 1 for “Yes” and 0 for “No.” The total scores for each subdomain were calculated by adding the scores of the questions. The total FC score range was 0–13; IADL, 0–5; intellectual activity, 0–4; and social role 0–4. If the TMIG-IC score in 2019 was lower than that in 2016, it was categorized as a decline. If the TMIG-IC score was equal to or higher than that in 2016, it was categorized as no decline. Functional decline was defined as a decrease of ≥1 point in TMIG-IC score during follow-up. This definition was adopted in a previous longitudinal study (13) to detect early deterioration in FC among community-dwelling older adults, as TMIG-IC is known to exhibit ceiling effects, and even small decreases may indicate early loss of FC.

#### 2.2.3 Covariates

Sex (male, female), age (65–69, 70–74, 75–79, 80–84, ≥85 years), number of teeth [0 (edentulous), 1–9, 10–19 teeth], educational level (<9, 10–12, ≥13 years), equivalent income (<1.00, 1.00–1.99, 2.00–2.99, 3.00–3.99, ≥4.00 million JPY), marital status (with a spouse, without a spouse), smoking status (current, former, never), history of alcohol consumption (current, former, never), and comorbidities (diabetes mellitus, hypertension, stroke) were included as covariates per previous studies (6). Equivalent income was calculated by dividing household income by the square root of the number of household members.

### 2.3 Statistical analysis

We conducted a modified Poisson regression analysis (14) with robust standard errors to estimate relative risks (RRs) and 95% confidence intervals (CIs). To reduce selection bias due to missing values, we conducted multiple imputations (MI) (15). We generated 20 imputed datasets using multivariate imputation by chained equations (MICE), including all variables used in the analysis. Subsequently, we combined the estimates obtained from each imputed dataset based on Rubin's rule. A complete case analysis was also conducted for sensitivity analysis. Additionally, several sensitivity analyses were conducted. First, we analyzed the use of each type of dental prosthesis (no use, removable dentures, fixed prostheses, implants, and using more than two types of dental prostheses) as an exposure variable. Second, we stratified the analysis according to the number of teeth (0–9 teeth vs. 10–19 teeth). As a sensitivity analysis, we additionally conducted analyses adjusting for baseline TMIG-IC scores. The results were generally consistent with the primary analysis; however, given the potential for bias in change-score analyses, these findings should be interpreted with caution and are presented as supplementary evidence of robustness (16). Statistical significance was set at *α* = 0.05. Stata/MP version 16.0 (Stata Corp.) was used for performing statistical analyses. We followed the Strengthening the Reporting of Observational Studies in Epidemiology (STROBE) guidelines (17).

### 2.4 Ethical approval

The JAGES in 2016 and 2019 were approved by the ethics committees of Nihon Fukushi University (No. 10-05), National Center for Geriatrics and Gerontology (No. 992, 1274-2), Chiba University (No. 2493, 3442), Tohoku University Graduate School of Dentistry (No. 37582), and the Japan Agency for Gerontological Evaluation Study (No. 2025-02). Informed consent was obtained from all the participants.

## 3 Results

A flowchart of participant inclusion is shown in Figure 1. Among 22,632 analyzed participants, the mean age was 74.0 years (SD = 5.7) in 2016, and 51.4% (*n* = 11,644) were men. The distribution of the participants' characteristics before MI is shown in Supplementary Table S1. The proportion of missing data for each variable ranged from 0.8% (marital and smoking status) to 19.6% (equivalent income). The participants' baseline characteristics after MI are presented in Table 1. The characteristics of the TMIG-IC total score and each domain score during the observation period are shown in Table 2. Consequently, no significant changes were observed in the distributions of the total score or the domain scores over the three-year follow-up period. Overall, 88.7% of the participants (*n* = 20,066) used dental prostheses, and 11.3% (*n* = 2,566) did not use dental prostheses at baseline. The proportion of patients with 10–19 teeth at baseline was 51.8% (*n* = 11,721), 1–9 teeth was 33.1% (*n* = 7,485), and zero teeth (edentulous) was 15.1% (*n* = 3,426). A total of 33.5% of the participants (*n* = 7,573) experienced a decline in total TMIG-IC scores during the follow-up. For each domain of TMIG-IC, 6.8% (*n* = 1,534) experienced a decline in IADL, 16.4% (*n* = 3,707) a decline in intellectual activity, and 26.1% (*n* = 5,900) a decline in social role in 2016 and 2019. The incidence of TMIG-IC decline in the total and domain scores was higher among participants without dental prostheses than among those who used dental prostheses (Table 3).

**Table 1:** Baseline characteristics of participants after multiple imputations (*n* = 22,632).

Variables	All participants (*n* = 22,632)		No dental prostheses use (*n* = 2,566)		Dental prostheses use (*n* = 20,066)	
	*n*	%	*n*	%	*n*	%
Number of teeth, teeth						
Edentulous	3,426	15.1	186	7.3	3,240	16.1
1–9	7,485	33.1	491	19.2	6,993	34.9
10–19	11,721	51.8	1,888	73.6	9,833	49.0
Sex						
Male	11,644	51.4	1,558	60.7	10,086	50.3
Female	10,988	48.6	1,008	39.3	9,980	49.7
Age, years						
65–69	6,173	27.3	1,039	40.5	5,134	25.6
70–74	6,692	29.6	784	30.5	5,909	29.4
75–79	5,730	25.3	490	19.1	5,240	26.1
80–84	2,961	13.1	188	7.3	2,773	13.8
≥85	1,076	4.8	66	2.6	1,010	5.0
Marital Status						
Without a spouse	5,895	26.0	695	27.1	5,200	25.9
With a spouse	16,737	74.0	1,871	72.9	14,866	74.1
Years of education, years						
<9	7,130	31.5	802	31.3	6,328	31.5
10–12	9,801	43.3	1,074	41.9	8,727	43.5
≥13	5,701	25.2	690	26.9	5,012	25.0
Equivalent income, million, JPY						
<1.00	2,842	12.6	370	14.4	2,472	12.3
1.00–1.99	8,684	38.4	1,022	39.8	7,662	38.2
2.00–2.99	5,481	24.2	599	23.3	4,882	24.3
3.00–3.99	3,288	14.5	338	13.2	2,950	14.7
≥4.00	2,337	10.3	238	9.3	2,099	10.5
Smoking Status						
Current Smoker	3,133	13.8	508	19.8	2,626	13.1
Former Smoker	7,464	33.0	791	30.8	6,673	33.3
Never Smoker	12,034	53.2	1,267	49.4	10,768	53.7
Alcohol consumption						
Current Drinker	9,226	40.8	1,150	44.8	8,076	40.2
Former Drinker	2,456	10.9	299	11.6	2,157	10.7
Never Drinker	10,950	48.4	1,118	43.5	9,833	49.0
Diabetes mellitus						
No	19,389	85.7	2,172	84.7	17,216	85.8
Yes	3,243	14.3	394	15.3	2,850	14.2
Hypertension						
No	12,309	54.4	1,429	55.7	10,879	54.2
Yes	10,324	45.6	1,137	44.3	9,187	45.8
Stroke						
No	22,026	97.3	2,476	96.5	19,549	97.4
Yes	606	2.7	90	3.5	517	2.6

Values are the average of 20 imputed datasets.

**Table 2:** Descriptive statistics for dental prostheses use and TMIG-IC scores in 2016 and 2019 (*n* = 22,632).

Variables	All participants (*n* = 22,632)	No dental prostheses use (*n* = 2,566)	Dental prostheses use (*n* = 20,066)
	Mean ± SD	Mean ± SD	Mean ± SD
Total			
2016	11.7 ± 1.6	11.4 ± 1.8	11.7 ± 1.5
2019	11.4 ± 1.9	11.1 ± 2.0	11.4 ± 1.8
IADL			
2016	4.9 ± 0.4	4.9 ± 0.5	4.9 ± 0.4
2019	4.8 ± 0.6	4.8 ± 0.7	4.8 ± 0.6
Intellectual activity			
2016	3.6 ± 0.7	3.5 ± 0.8	3.6 ± 0.7
2019	3.5 ± 0.8	3.4 ± 0.9	3.5 ± 0.8
Social role			
2016	3.2 ± 1.0	3.0 ± 1.1	3.2 ± 1.0
2019	3.1 ± 1.1	2.9 ± 1.2	3.1 ± 1.1

**Table 3:** Proportion of TMIG-IC decline by dental prosthesis use (*n* = 22,632).

Outcome variables	All participants (*n* = 22,632)		No dental prostheses use (*n* = 2,566)		Dental prostheses use (*n* = 20,066)	
	*n*	%	*n*	%	*n*	%
Total						
Not declined	15,059	66.5	1,647	64.2	13,412	66.8
Declined	7,573	33.5	919	35.8	6,654	33.2
IADL						
Not declined	21,098	93.2	2,391	93.2	18,707	93.2
Declined	1,534	6.8	175	6.8	1,359	6.8
Intellectual activity						
Not declined	18,925	83.6	2,090	81.4	16,835	83.9
Declined	3,707	16.4	476	18.6	3,231	16.1
Social role						
Not declined	16,732	73.9	1,872	72.9	14,861	74.1
Declined	5,900	26.1	695	27.1	5,205	25.9

IADL, instrumental activities of daily living.

The modified Poisson regression model is presented in Table 4. In the adjusted models, dental prostheses were significantly associated with a lower risk of decline in the total TMIG-IC score [RR = 0.91 (95% CI: 0.86–0.97)] compared with that with no use. For the TMIG-IC subdomain, we also observed a significant association between dental prosthesis use and a lower risk of decline in intellectual activity [RR = 0.87 (95% CI: 0.80–0.96)]. However, significant association was not observed for IADL [RR = 0.88 (95% CI: 0.75–1.03)] and social role [RR = 0.95 (95% CI: 0.88–1.02)]. Dental prostheses were associated with approximately a 9% lower risk of decline in total FC and a 13% lower risk of decline in intellectual activity among older adults. The findings of the complete case analysis also suggested results similar to those derived from the MI datasets (Supplementary Table S2).

**Table 4:** Association between prosthesis use and decline of functional capacity (*n* = 22,632).

	Crude model		Model 1		Model 2	
	RR (95% CI)	*P*	RR (95% CI)	*P*	RR (95% CI)	*P*
Total						
No dental prostheses use	1 (Ref.)		1 (Ref.)		1 (Ref.)	
Dental prostheses Use	0.93 (0.88, 0.98)	0.007	0.91 (0.86, 0.97)	0.002	0.91 (0.86, 0.97)	0.002
IADL						
No dental prostheses use	1 (Ref.)		1 (Ref.)		1 (Ref.)	
Dental prostheses Use	0.99 (0.85, 1.15)	0.906	0.91 (0.78, 1.06)	0.222	0.88 (0.75, 1.03)	0.104
Intellectual activity						
No dental prostheses use	1 (Ref.)		1 (Ref.)		1 (Ref.)	
Dental prostheses Use	0.87 (0.80, 0.95)	<0.001	0.89 (0.81, 0.97)	0.008	0.87 (0.80, 0.96)	0.003
Social role						
No dental prostheses use	1 (Ref.)		1 (Ref.)		1 (Ref.)	
Dental prostheses Use	0.96 (0.90, 1.03)	0.222	0.94 (0.88, 1.01)	0.071	0.95 (0.88, 1.02)	0.136

CI, Confidence interval; IADL, instrumental activities of daily living; RR, Relative risk.

Model 1 was adjusted for sex and age. Model 2 was adjusted for sex, age, number of teeth, educational level, equivalent income (JPY), marital status, smoking status, history of alcohol consumption, and comorbidities (diabetes mellitus, hypertension, and stroke). Prostheses include removable dentures, fixed prostheses, and implants.

We performed several sensitivity analyses. First, we stratified participants by the number of teeth into two groups: 0–9 or 10–19 teeth (Supplementary Table S3). Similar point estimates were obtained from the main analysis for both strata. Second, we used the types of dental prostheses as exposures (no use, removable dentures, fixed prostheses, implants, and use of more than two types of dental prostheses) to evaluate the differences in the association between TMIG-IC decline and the types of dental prostheses (Supplementary Table S4). In line with the primary analysis, for the use of removable dentures, significant associations were observed between the total TMIG-IC score and intellectual activity. The group with the lowest risk of a decline in intellectual activity was not the one using fixed prostheses [RR = 0.93 (95% CI: 0.81–1.07)] or implants alone [RR = 0.88 (95% CI: 0.67–1.15)] but rather the one using removable dentures [RR = 0.87 (95% CI: 0.79–0.95)] and a combination of two or more types of dental prostheses [RR = 0.85 (95% CI: 0.74–0.99)]. Sensitivity analysis adjusted for baseline TMIG-IC scores also showed robustness of the results (Supplementary Table S5).

## 4 Discussion

In this large-scale prospective cohort study of older adults with tooth loss, dental prosthesis use was associated with a significantly lower risk of total FC decline, as measured by the TMIG-IC score, over a three-year period. Notably, this protective association was significant for the intellectual activity domain, whereas associations with IADL and social role were not statistically significant. These findings were consistent across multiple sensitivity analyses, including stratification according to the number of teeth and types of dental prostheses. Specifically, the use of removable dentures alone or with multiple types of dental prostheses was associated with a reduced risk of decline in intellectual activity, suggesting that the type and combination of prosthetic rehabilitation may have different effects across FC domains.

Our findings are consistent with those of previous studies that reported an association between tooth loss and a decline in FC among older adults. Sato et al. (6) demonstrated that tooth loss was linked to an increased risk of total functional decline, as measured by TMIG-IC scores, and Tsakos et al. (18) similarly reported associations between tooth loss and both physical and cognitive decline in a cohort of older adults in England. Building on these findings, the present study extends prior work by focusing on dental prosthesis use among individuals with tooth loss and by examining domain-specific components of FC using longitudinal cohort data.

The observed association between dental prosthesis use and reduced risk of decline in intellectual activity is also consistent with those of previous studies showing that prosthetic rehabilitation may help preserve cognitive function. For example, a 7-year longitudinal study of over 64,000 Taiwanese older adults reported that increased functional tooth count—through the acquisition of removable dentures, fixed prostheses, or implants—was associated with a reduced risk of cognitive impairment (19). Similarly, findings from the Chinese Longitudinal Healthy Longevity Survey showed that denture use moderated the negative impact of partial tooth loss on cognitive decline, particularly among those with <20 teeth (20). A potential underlying mechanism is that denture use may improve masticatory function and masseter muscle activity, thereby enhancing brain function in domains such as attention, verbal skills, and visual memory (21). This neuroimaging study has suggested that mastication stimulates multiple brain regions, including gyrus and cerebellum, which are involved in cognitive processes. Although the mechanisms remain incompletely understood, restoration of masticatory ability resulting from prosthodontic rehabilitation may therefore contribute to maintaining intellectual activity in later life.

Conversely, our analysis did not find a significant association between dental prosthesis use and a decline in IADL or social role. However, the stratified analyses suggested a potential benefit of IADL among participants with 0–9 remaining teeth. The restricted analytical sample size of individuals with <20 teeth may explain the lack of significant associations in these domains. In the older study population, the baseline levels of IADL and social role may be relatively low, limiting the detectable effects of dental prosthesis use. Previous studies using reference groups with ≥20 teeth may have achieved a greater contrast in outcomes, highlighting the benefit of dental prostheses by comparison (6, 22, 23). Our findings complement existing literature by specifically focusing on a population (participants with <20 teeth) that is thought to benefit most from prosthodontic intervention.

To the best of our knowledge, this study is the first to evaluate the association between dental prosthesis use and the individual components of FC decline. By limiting our analytical sample to individuals with fewer than 20 teeth, we minimized the confounding effects of natural dentition, which is independently associated with better functional outcomes (6, 22, 23). This design enabled a more precise evaluation of dental prosthesis effects, independent of the influence of preserved natural teeth.

These findings contribute to a growing body of evidence highlighting the importance of oral health in maintaining functional ability in aging populations. As the global population continues to age, preserving FC among older adults is a critical goal (24). FC decline is influenced not only by structural degeneration but also by modifiable functional changes associated with disuse or early stage frailty (25). Consequently, FC represents a dynamic and potentially reversible outcome that can be targeted through timely and appropriate intervention (26).

In the present study, stratified analyses by type of dental prosthesis showed that, as in the primary analysis, significant associations were observed between the use of removable dentures and TMIG-IC total score and intellectual activity. Interestingly, the group with the lowest risk of decline in intellectual activity was not those using fixed prostheses, suggesting that prosthesis type may differentially influence functional outcomes (Supplementary Table S4). These findings indicate that maintaining oral function through prosthodontic rehabilitation, particularly through the use of removable dentures, may help support both cognitive and functional aspects of FC in aging populations.

This perspective aligns with the growing recognition of oral frailty as a transitional state characterized by cumulative decline in multiple oral functions, including mastication, swallowing, and articulation. Oral frailty has been associated not only with physical and cognitive decline (27) but also with undernutrition and an increased risk of disability (28, 29). Although the present study did not directly assess oral frailty using established screening tools such as the 5-item Oral Frailty Checklist (OF-5) (28), the concept of declining oral FC overlaps with the broader framework of oral frailty. Maintaining oral function through appropriate prosthodontic rehabilitation may therefore represent one potential approach for supporting functional health in aging populations.

Several limitations should be considered when interpreting the findings. First, although dental prosthesis use was assessed before the occurrence of functional decline, the possibility of reverse causality cannot be completely excluded. Individuals with better baseline, prosthesis use was categorized into simplified groups, FC may have been more likely to obtain or maintain dental prostheses, which could partly explain the observed association.

Second, dental prosthesis use was assessed using self-reported questionnaire data and did not include objective clinical assessments of prosthetic fit, functional performance, or patterns of daily use. In addition, prosthesis use was measured only at baseline, and changes in prosthetic status during the follow-up period could not be captured. Such exposure misclassification is likely to be nondifferential and may have attenuated the observed associations (30).

Third, FC was measured using the TMIG-IC, a self-administered questionnaire. Although this instrument has been widely used and validated for assessing FC among community-dwelling older adults in Japan (3, 31), self-reported measures may be influenced by subjective perception or reporting bias. In addition, functional decline was defined as a decrease in TMIG-IC score during follow-up and was analyzed as a binary outcome in this study. This approach facilitates the identification of incident functional decline but may not fully capture the magnitude or trajectory of functional change. Moreover, the simplified measurement of both exposure and outcome may have introduced measurement error. The use of a dichotomized outcome may have attenuated or obscured more nuanced associations, and these limitations should be considered when interpreting the findings as associations rather than causal effects.

Fourth, selection bias due to attrition during follow-up may have influenced the results. Older adults with poorer oral health or lower FC may have been more likely to drop out of the cohort because of death, institutionalization, or the onset of disability. If participants with poorer health conditions were more likely to be lost to follow-up, the association between dental prosthesis use and FC decline may have been underestimated. However, part of the loss to follow-up in the JAGES cohort was related to survey design in some municipalities, where follow-up questionnaires were distributed to randomly selected residents rather than to all baseline participants. This mechanism may have reduced the likelihood of systematic bias. Additionally, although attrition bias may have affected the results, previous methodological studies suggest that such bias is unlikely to substantially change the direction of associations or overall conclusions (32).

Finally, although we adjusted for a range of sociodemographic and health-related factors, the possibility of residual confounding by unmeasured factors cannot be ruled out. Further studies incorporating more detailed clinical and behavioral information would help clarify the mechanisms underlying the observed associations.

This study also has several strengths. It used data from a large-scale cohort of community-dwelling older adults in Japan and allowed detailed examination of both exposure and outcome variables. Specifically, dental prosthesis use was categorized by type of prosthesis, and FC was evaluated across multiple domains, including IADL, intellectual activity, and social role. These features enabled a nuanced analysis of the association between prosthetic rehabilitation and different aspects of FC decline.

## 5 Conclusions

This large-scale cohort study demonstrated that the use of dental prostheses was associated with a lower risk of FC decline among older adults, particularly in the domain of intellectual activity. These findings suggest an association between prosthodontic rehabilitation and the maintenance of cognitive and functional abilities later in life. However, given these findings should be interpreted in light of the observational design and the use of simplified, self-reported measures for both exposure and outcome. Future longitudinal studies with more detailed clinical assessments and intervention-based designs are warranted to clarify the underlying mechanisms and potential the causal pathways between oral rehabilitation and preservation of FC in aging population.

## Data Availability

The datasets presented in this article are not readily available because Data were obtained from the JAGES study. All JAGES datasets have ethical or legal restrictions for public deposition owing to the inclusion of sensitive information from human participants. Requests to access the datasets should be directed to JAGES Data Management Committee, dataadmin.ml@jages.net.
