# Supplementary material for: Dental prosthesis use and subsequent functional capacity decline among older adults: a three-year cohort study based on self-reported measures from the JAGES
Source: Front Oral Health. 2026 May 7;7:1784871. doi: 10.3389/froh.2026.1784871 (PMC13190564; doi:10.3389/froh.2026.1784871)
Supplement: Supplementary file 1 [file Supplementaryfile1.docx]

Supplementary Material

# Supplementary Text: Questionnaire of the Tokyo Metropolitan Institute of Gerontology Index of Competence (TMIG-IC).

Instrumental Self-Maintenance

(1) Can you use public transportation (bus or train) by yourself?

(2) Are you able to shop for daily necessities?

(3) Are you able to prepare meals by yourself?

(4) Are you able to pay bills?

(5) Can you handle your own bank?

Intellectual Activity

(6) Are you able to fill in the form for your pension?

(7) Do you read newspapers?

(8) Do you read books or magazines?

(9) Are you interested in news stories or programs dealing with health?

Social Role

(10) Do you visit your friends’ homes?

(11) Are you sometimes called on for advice?

(12) Are you able to visit sick friends?

(13) Do you sometimes initiate conversations with young people?

# Supplementary Table 1: Baseline characteristics of participants before multiple imputations (n = 22,632).

| Variables |  | All participants (n = 22,632) | | No dental prostheses use (n = 2,543) | | Dental prostheses use (n = 19,914) | | Missing value  (n = 175) | |
| --- | --- | --- | --- | --- | --- | --- | --- | --- | --- |
|  |  | n | % | n | % | n | % | n | % |
| Number of teeth, teeth |  |  |  |  |  |  |  |  |  |
|  | Edentulous | 3,334 | 14.7 | 179 | 7.0 | 3,140 | 15.8 | 15 | 8.6 |
|  | 1–9 | 7,286 | 32.2 | 467 | 18.4 | 6,798 | 34.1 | 21 | 12.0 |
|  | 10–19 | 11,375 | 50.3 | 1,790 | 70.4 | 9,492 | 47.7 | 93 | 53.1 |
|  | Missing value | 637 | 2.8 | 107 | 4.2 | 484 | 2.4 | 46 | 26.3 |
| Sex |  |  |  |  |  |  |  |  |  |
|  | Male | 11,644 | 51.4 | 1,549 | 60.9 | 10,034 | 50.4 | 61 | 34.9 |
|  | Female | 10,988 | 48.6 | 994 | 39.1 | 9,880 | 49.6 | 114 | 65.1 |
| Age, years |  |  |  |  |  |  |  |  |  |
|  | 65–69 | 6,173 | 27.3 | 1,032 | 40.6 | 5,103 | 25.6 | 38 | 21.7 |
|  | 70–74 | 6,692 | 29.6 | 776 | 30.5 | 5,868 | 29.5 | 48 | 27.4 |
|  | 75–79 | 5,730 | 25.3 | 484 | 19.0 | 5,192 | 26.1 | 54 | 30.9 |
|  | 80–84 | 2,961 | 13.1 | 186 | 7.3 | 2,749 | 13.8 | 26 | 14.9 |
|  | ≥85 | 1,076 | 4.8 | 65 | 2.6 | 1,002 | 5.0 | 9 | 5.1 |
| Marital Status |  |  |  |  |  |  |  |  |  |
|  | Without a spouse | 5,838 | 25.8 | 680 | 26.7 | 5,107 | 25.6 | 51 | 29.1 |
|  | With a spouse | 16,623 | 73.4 | 1,841 | 72.4 | 14,660 | 73.6 | 122 | 69.7 |
|  | Missing value | 171 | 0.8 | 22 | 0.9 | 147 | 0.7 | 2 | 1.1 |
| Years of education, years | |  |  |  |  |  |  |  |  |
|  | <9 | 7,040 | 31.1 | 780 | 30.7 | 6,173 | 31.0 | 87 | 49.7 |
|  | 10–12 | 9,697 | 42.8 | 1,058 | 41.6 | 8,584 | 43.1 | 55 | 31.4 |
|  | ≥13 | 5,649 | 25.0 | 681 | 26.8 | 4,940 | 24.8 | 28 | 16.0 |
|  | Missing value | 246 | 1.1 | 24 | 0.9 | 217 | 1.1 | 5 | 2.9 |
| Equivalent income, million, JPY | |  |  |  |  |  |  |  |  |
|  | <1.00 | 2,198 | 9.7 | 293 | 11.5 | 1,874 | 9.4 | 31 | 17.7 |
|  | 1.00–1.99 | 6,887 | 30.4 | 830 | 32.6 | 6,012 | 30.2 | 45 | 25.7 |
|  | 2.00–2.99 | 4,459 | 19.7 | 488 | 19.2 | 3,949 | 19.8 | 22 | 12.6 |
|  | 3.00–3.99 | 2,720 | 12.0 | 280 | 11.0 | 2,431 | 12.2 | 9 | 5.1 |
|  | ≥4.00 | 1,939 | 8.6 | 198 | 7.8 | 1,731 | 8.7 | 10 | 5.7 |
|  | Missing value | 4,429 | 19.6 | 454 | 17.9 | 3,917 | 19.7 | 58 | 33.1 |
| Smoking status |  |  |  |  |  |  |  |  |  |
|  | Current smoker | 3,121 | 13.8 | 504 | 19.8 | 2,599 | 13.1 | 18 | 10.3 |
|  | Former smoker | 7,427 | 32.8 | 784 | 30.8 | 6,614 | 33.2 | 29 | 16.6 |
|  | Never smoker | 11,913 | 52.6 | 1,241 | 48.8 | 10,547 | 53.0 | 125 | 71.4 |
|  | Missing value | 171 | 0.8 | 14 | 0.6 | 154 | 0.8 | 3 | 1.7 |
| Alcohol consumption |  |  |  |  |  |  |  |  |  |
|  | Current drinker | 9,131 | 40.3 | 1,133 | 44.6 | 7,952 | 39.9 | 46 | 26.3 |
|  | Former drinker | 2,422 | 10.7 | 294 | 11.6 | 2,117 | 10.6 | 11 | 6.3 |
|  | Never drinker | 10,738 | 47.4 | 1,083 | 42.6 | 9,543 | 47.9 | 112 | 64.0 |
|  | Missing value | 341 | 1.5 | 33 | 1.3 | 302 | 1.5 | 6 | 3.4 |
| Diabetes mellitus |  |  |  |  |  |  |  |  |  |
|  | No | 18,798 | 83.1 | 2,089 | 82.1 | 16,567 | 83.2 | 142 | 81.1 |
|  | Yes | 3,153 | 13.9 | 379 | 14.9 | 2,753 | 13.8 | 21 | 12.0 |
|  | Missing value | 681 | 3.0 | 75 | 2.9 | 594 | 3.0 | 12 | 6.9 |
| Hypertension |  |  |  |  |  |  |  |  |  |
|  | No | 11,935 | 52.7 | 1,374 | 54.0 | 10,473 | 52.6 | 88 | 50.3 |
|  | Yes | 10,016 | 44.3 | 1,094 | 43.0 | 8,847 | 44.4 | 75 | 42.9 |
|  | Missing value | 681 | 3.0 | 75 | 2.9 | 594 | 3.0 | 12 | 6.9 |
| Stroke |  |  |  |  |  |  |  |  |  |
|  | No | 21,361 | 94.4 | 2,381 | 93.6 | 18,820 | 94.5 | 160 | 91.4 |
|  | Yes | 590 | 2.6 | 87 | 3.4 | 500 | 2.5 | 3 | 1.7 |
|  | Missing value | 681 | 3.0 | 75 | 2.9 | 594 | 3.0 | 12 | 6.9 |

# Supplementary Table 2: Association between prosthesis use and functional capacity decline in complete case analysis (n = 16,760).

|  | | Crude Model | | Model 1 | | Model 2 | |
| --- | --- | --- | --- | --- | --- | --- | --- |
|  |  | RR (95% CI) | *P* | RR (95% CI) | *P* | RR (95% CI) | *P* |
| Total |  |  |  |  |  |  |  |
|  | No dental prostheses use | 1 (Ref.) |  | 1 (Ref.) |  | 1 (Ref.) |  |
|  | Dental prostheses use | 0.93 (0.87, 0.99) | 0.027 | 0.92 (0.86, 0.98) | 0.013 | 0.93 (0.87, 0.99) | 0.020 |
| IADL |  |  |  |  |  |  |  |
|  | No dental prostheses use | 1 (Ref.) |  | 1 (Ref.) |  | 1 (Ref.) |  |
|  | Dental prostheses use | 0.95 (0.80, 1.14) | 0.589 | 0.88 (0.74, 1.05) | 0.165 | 0.86 (0.72, 1.03) | 0.109 |
| Intellectual activity | |  |  |  |  |  |  |
|  | No dental prostheses use | 1 (Ref.) |  | 1 (Ref.) |  | 1 (Ref.) |  |
|  | Dental prostheses use | 0.88 (0.80, 0.98) | 0.015 | 0.91 (0.82, 1.01) | 0.061 | 0.90 (0.81, 0.99) | 0.038 |
| Social role | |  |  |  |  |  |  |
|  | No dental prostheses use | 1 (Ref.) |  | 1 (Ref.) |  | 1 (Ref.) |  |
|  | Dental prostheses use | 0.94 (0.87, 1.02) | 0.143 | 0.93 (0.86, 1.01) | 0.065 | 0.94 (0.87, 1.02) | 0.125 |

CI, Confidence interval; IADL, instrumental activities of daily living; RR, Relative risk.

Model 1 was adjusted for sex and age. Model 2 was adjusted for sex, age, number of teeth, educational level, equivalent income (JPY), marital status, smoking status, history of alcohol consumption, and comorbidities (diabetes mellitus, hypertension, and stroke). Prostheses included removable dentures, fixed prostheses, and implants.

# Supplementary Table 3: Association between prosthesis use and decline of functional capacity with teeth categories among all participants (n = 22,632).

|  |  | 10–19 teeth (n = 11,721) | | 0–9 teeth (n = 10,911) | |
| --- | --- | --- | --- | --- | --- |
|  |  | RR (95% CI) | *P* | RR (95% CI) | *P* |
| Total |  |  |  |  |  |
|  | No dental prostheses use | 1 (Ref.) |  | 1 (Ref.) |  |
|  | Dental prostheses use | 0.92 (0.86, 0.99) | 0.024 | 0.91 (0.82, 1.01) | 0.071 |
| IADL |  |  |  |  |  |
|  | No dental prostheses use | 1 (Ref.) |  | 1 (Ref.) |  |
|  | Dental prostheses use | 0.94 (0.77, 1.15) | 0.548 | 0.78 (0.61, 1.00) | 0.047 |
| Intellectual activity |  |  |  |  |  |
|  | No dental prostheses use | 1 (Ref.) |  | 1 (Ref.) |  |
|  | Dental prostheses use | 0.86 (0.77, 0.96) | 0.009 | 0.87 (0.75, 1.01) | 0.071 |
| Social role |  |  |  |  |  |
|  | No dental prostheses use | 1 (Ref.) |  | 1 (Ref.) |  |
|  | Dental prostheses use | 0.95 (0.87, 1.04) | 0.257 | 0.95 (0.84, 1.09) | 0.470 |

CI, Confidence interval; IADL, instrumental activities of daily living; RR, Relative risk.

Model was adjusted for sex, age, number of teeth, educational level, equivalent income (JPY), marital status, smoking status, history of alcohol consumption, and comorbidities (diabetes mellitus, hypertension, and stroke). Prostheses included removable dentures, fixed prostheses, and implants.

# Supplementary Table 4: Association between prosthetic use and TMIG-IC between 2016-19 after multiple imputations (n = 22,632).

|  |  | Model 1 | | Model 2 | |
| --- | --- | --- | --- | --- | --- |
|  |  | RR (95% CI) | *P* | RR (95% CI) | *P* |
| Total |  |  |  |  |  |
|  | No use | 1 (Ref.) |  | 1 (Ref.) |  |
|  | Removable dentures only | 0.91 (0.86, 0.97) | <.001 | 0.90 (0.85, 0.96) | <.001 |
|  | Fixed prostheses only | 0.92 (0.84, 1.00) | 0.063 | 0.94 (0.86, 1.03) | 0.162 |
|  | Implants only | 0.94 (0.80, 1.10) | 0.450 | 0.99 (0.84, 1.16) | 0.895 |
|  | More than 2 types | 0.93 (0.85, 1.01) | 0.090 | 0.95 (0.87, 1.04) | 0.274 |
| IADL |  |  |  |  |  |
|  | No use | 1 (Ref.) |  | 1 (Ref.) |  |
|  | Removable dentures only | 0.92 (0.79, 1.07) | 0.279 | 0.87 (0.74, 1.02) | 0.076 |
|  | Fixed prostheses only | 0.93 (0.73, 1.19) | 0.565 | 0.99 (0.77, 1.27) | 0.919 |
|  | Implants only | 0.70 (0.42, 1.18) | 0.183 | 0.78 (0.47, 1.31) | 0.353 |
|  | More than 2 types | 0.84 (0.66, 1.07) | 0.152 | 0.90 (0.70, 1.15) | 0.393 |
| Intellectual activity |  |  |  |  |  |
|  | No use | 1 (Ref.) |  | 1 (Ref.) |  |
|  | Removable dentures only | 0.90 (0.82, 0.99) | 0.023 | 0.87 (0.79, 0.95) | 0.003 |
|  | Fixed prostheses only | 0.87 (0.76, 1.01) | 0.063 | 0.93 (0.81, 1.07) | 0.300 |
|  | Implants only | 0.79 (0.61, 1.03) | 0.084 | 0.88 (0.67, 1.15) | 0.337 |
|  | More than 2 types | 0.80 (0.69, 0.92) | 0.002 | 0.85 (0.74, 0.99) | 0.031 |
| Social role |  |  |  |  |  |
|  | No use | 1 (Ref.) |  | 1 (Ref.) |  |
|  | Removable dentures only | 0.94 (0.87, 1.00) | 0.062 | 0.94 (0.88, 1.01) | 0.102 |
|  | Fixed prostheses only | 0.92 (0.82, 1.02) | 0.126 | 0.93 (0.83, 1.04) | 0.188 |
|  | Implants only | 1.01 (0.84, 1.21) | 0.950 | 1.06 (0.88, 1.28) | 0.527 |
|  | More than 2 types | 0.97 (0.88, 1.08) | 0.596 | 1.00 (0.90, 1.11) | 0.937 |

CI, Confidence interval; IADL, instrumental activities of daily living; RR, Relative risk.

Model 1 was adjusted for sex and age. Model 2 was adjusted for sex, age, number of teeth, educational level, equivalent income (JPY), marital status, smoking status, history of alcohol consumption, and comorbidities (diabetes mellitus, hypertension, and stroke). Prostheses included removable dentures, fixed prostheses, and implants.

**Supplementary Table 5:** Association between prosthesis use and decline of functional capacity with adjustment for baseline scores (n = 22,632).

|  |  | Crude model | | Model 1 | | Model 2 | |
| --- | --- | --- | --- | --- | --- | --- | --- |
|  |  | RR (95% CI) | *P* | RR (95% CI) | *P* | RR (95% CI) | *P* |
| Total |  |  |  |  |  |  |  |
|  | No dental prostheses use | 1 (Ref.) |  | 1 (Ref.) |  | 1 (Ref.) |  |
|  | Dental prostheses Use | 0.91 (0.86, 0.97) | 0.001 | 0.90 (0.85, 0.95) | <0.001 | 0.90 (0.85, 0.95) | <0.001 |
| IADL |  |  |  |  |  |  |  |
|  | No dental prostheses use | 1 (Ref.) |  | 1 (Ref.) |  | 1 (Ref.) |  |
|  | Dental prostheses Use | 1.02 (0.88, 1.19) | 0.775 | 0.94 (0.80, 1.09) | 0.388 | 0.90 (0.75, 1.03) | 0.178 |
| Intellectual activity | |  |  |  |  |  |  |
|  | No dental prostheses use | 1 (Ref.) |  | 1 (Ref.) |  | 1 (Ref.) |  |
|  | Dental prostheses Use | 0.86 (0.78, 0.93) | <0.001 | 0.88 (0.80, 0.96) | 0.003 | 0.86 (0.78, 0.94) | 0.001 |
| Social role | |  |  |  |  |  |  |
|  | No dental prostheses use | 1 (Ref.) |  | 1 (Ref.) |  | 1 (Ref.) |  |
|  | Dental prostheses Use | 0.93 (0.87, 1.00) | 0.039 | 0.91 (0.85, 0.98) | 0.008 | 0.92 (0.86, 0.99) | 0.019 |

CI, Confidence interval; IADL, instrumental activities of daily living; RR, Relative risk.

Model 1 was adjusted for each TMIG-IC score. Model 2 was adjusted for sex, age, and each TMIG-IC score. Model 3 was adjusted for sex, age, number of teeth, educational level, equivalent income (JPY), marital status, smoking status, history of alcohol consumption, comorbidities (diabetes mellitus, hypertension, and stroke), and each TMIG-IC score. Prostheses include removable dentures, fixed prostheses, and implants.
